# Natural history of a bighorn sheep pneumonia epizootic: Source of infection, course of disease, and pathogen clearance

**DOI:** 10.1002/ece3.8166

**Published:** 2021-09-23

**Authors:** Thomas E. Besser, E. Frances Cassirer, Amy Lisk, Danielle Nelson, Kezia R. Manlove, Paul C. Cross, John T. Hogg

**Affiliations:** ^1^ Department of Veterinary Microbiology and Pathology Washington State University Pullman Washington USA; ^2^ Idaho Department of Fish and Game Lewiston Idaho USA; ^3^ US Fish and Wildlife Service Moiese Montana USA; ^4^ Washington Animal Disease Diagnostic Laboratory Department of Veterinary Microbiology and Pathology Washington State University Pullman Washington USA; ^5^ Department of Wildland Resources & Ecology Center Utah State University Logan Utah USA; ^6^ U. S. Geological Survey Northern Rocky Mountain Science Center Bozeman Montana USA; ^7^ Montana Conservation Science Institute Missoula Montana USA

**Keywords:** bighorn sheep, domestic sheep, *Mycoplasma ovipneumoniae*, respiratory disease, spillover, wildlife–livestock interface

## Abstract

A respiratory disease epizootic at the National Bison Range (NBR) in Montana in 2016–2017 caused an 85% decline in the bighorn sheep population, documented by observations of its unmarked but individually identifiable members, the subjects of an ongoing long‐term study. The index case was likely one of a small group of young bighorn sheep on a short‐term exploratory foray in early summer of 2016. Disease subsequently spread through the population, with peak mortality in September and October and continuing signs of respiratory disease and sporadic mortality of all age classes through early July 2017. Body condition scores and clinical signs suggested that the disease affected ewe groups before rams, although by the end of the epizootic, ram mortality (90% of 71) exceeded ewe mortality (79% of 84). Microbiological sampling 10 years to 3 months prior to the epizootic had documented no evidence of infection or exposure to *Mycoplasma ovipneumoniae* at NBR, but during the epizootic, a single genetic strain of *M. ovipneumoniae* was detected in affected animals. Retrospective screening of domestic sheep flocks near the NBR identified the same genetic strain in one flock, presumptively the source of the epizootic infection. Evidence of fatal lamb pneumonia was observed during the first two lambing seasons following the epizootic but was absent during the third season following the death of the last identified *M. ovipneumoniae* carrier ewe. Monitoring of life‐history traits prior to the epizootic provided no evidence that environmentally and/or demographically induced nutritional or other stress contributed to the epizootic. Furthermore, the epizootic occurred despite proactive management actions undertaken to reduce risk of disease and increase resilience in this population. This closely observed bighorn sheep epizootic uniquely illustrates the natural history of the disease including the (presumptive) source of spillover, course, severity, and eventual pathogen clearance.

## Graphical abstract

This case report provides unprecedentedly detailed animal observations before, during, and after a severe bighorn sheep pneumonia outbreak and includes molecular detection of the causative primary pathogen, *Mycoplasma ovipneumoniae*, in its putative source, a nearby domestic sheep operation. As a result, this case report serves to advance our understanding of critical aspects of this important disease.

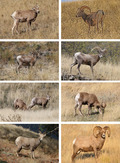

## 1 INTRODUCTION

Epizootic pneumonia of bighorn sheep is a devastating disease that contributed to the dramatic decline of this species following European settlement of the western United States and that today remains a major factor limiting the recovery of wild sheep (Cassirer et al., 2018). Two classes of hypotheses vie for acceptance as an explanation for this disease: Broad “epidemiological triad” hypotheses attribute bighorn sheep pneumonia epizootics to changes in or interactions among host, environment, and pathogen factors that predispose bighorn sheep to disease (Butler et al., 2018; Rachowicz et al., 2005). As a result, thorough testing of epidemiologic triad hypotheses requires consideration of a large number of host and environmental factors and their interactions, in addition to both preexisting and novel pathogens (Miller, Hoberg, et al., 2012; Monello et al., 2001; Sells et al., 2015). “Spillover” hypotheses are a subset of epidemiologic triad hypotheses involving “novel” pathogens where the hosts, due to lack of previous exposure and adaptation, may experience disease epizootics without significant or detectable changes in environmental or host factors. Spillover infections acquired from wild or domestic animal hosts are recognized as the cause of most emerging human infectious diseases (Jones et al., 2008). Similarly, many infectious diseases in wildlife are recognized to be a direct consequence of exposure to novel pathogens, including those transmitted from sympatric domestic animal reservoirs (Daszak et al., 2000). “Spillover” hypotheses for epizootic bighorn sheep pneumonia were first proposed very early in response to disease events following observed contacts with domestic sheep in captivity and in the wild (McCann, 1956; Shillinger, 1937). Considerable recent evidence supports the bacterium *Mycoplasma ovipneumoniae* as the primary spillover pathogen triggering epizootic bighorn sheep pneumonia (Besser, Cassirer, et al., 2012; Besser, Highland, et al., 2012; Cassirer et al., 2018; Kamath et al., 2019).

Empirical data on cross‐species pathogen spillover, disease emergence, and pathogen persistence are lacking and difficult to come by (Viana et al., 2014). We describe here detailed evidence from the 2016–2017 National Bison Range (NBR) epizootic that is entirely consistent with the *M. ovipneumoniae* spillover paradigm, including (a) identification of the time window for pathogen spillover and animal movements likely responsible for the spillover event, (b) precise enumeration of animal losses during the ensuing epizootic, (c) documentation of within‐population paths of pathogen transmission and eventual pathogen clearance, the latter of which was accompanied by fadeout of respiratory disease, and (d) identification of a local domestic sheep flock as the presumptive source for the single genetic strain of *M. ovipneumoniae* found in afflicted NBR bighorn sheep.

## 2 MATERIALS AND METHODS

### 2.1 Study site and population

**FIGURE 1:**
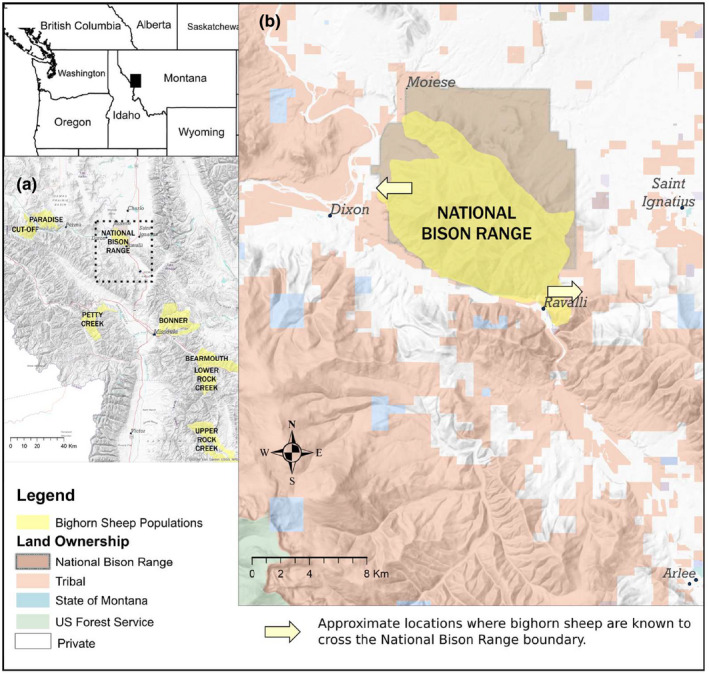
National Bison Range bighorn sheep study site. (a) Location of the National Bison Range (within dotted rectangle) and adjacent bighorn sheep populations (yellow polygons) in western Montana. (b) Close up of the distribution of the bighorn sheep population on the National Bison Range and adjacent lands within the Flathead Reservation of the Confederated Salish and Kootenai Tribes

The NBR is a 7,500 ha National Wildlife Refuge located in western Montana, USA, within the boundaries of the Flathead Reservation of the Confederated Salish and Kootenai Tribes (CSKT) (Figure 1). The refuge was formally transferred from the U. S. Fish and Wildlife Service (USFWS) to the CSKT by an act of Congress in January 2021. However, because all data reported here were collected prior to 2021 under permits issued by the USFWS, we refer to the study site in terms of its pretransfer management status.

The NBR bighorn sheep population was established in February 1922 by translocation of four males and eight females from Banff National Park (Alberta, Canada). There is no post‐1922 record in refuge annual reports or genetic evidence (Electronic Supplemental Material figure A1 in Hogg et al., 2006) of artificial or natural immigration, respectively, before 1985, when five rams were introduced. During 1990–1994, the population was supplemented with an additional 10 bighorn sheep (three male and seven female) from Montana and Wyoming. Subsequent admixture markedly increased genetic diversity, coincided with large rebounds in a broad range of phenotypic traits (Hogg et al., 2006; Miller, Poissant, et al., 2012), and was associated with a lagged demographic response in which the end‐of‐year population increased from a preadmixture (1979–1985) average of 42 to 205 animals in fall 2015 (Hogg et al., 2017). The resident bighorn sheep population has been the subject of continuous behavioral, ecological, and genetic study since 1979 (Hogg et al., 2017). Study animals are tolerant of human observers, and, since 1979, the entire adult population has been individually recognizable from ear notches applied during hand capture of neonates or by natural variations in horns and pelage.

The NBR is fenced along its perimeter (Figure 1b), but this fence is permeable to bighorn sheep. Continued genetic and demographic monitoring of resident bighorn sheep detected three natural immigrants to NBR following the experimental 1985–1994 introductions. These animals were first identified genetically as migrants in 2009, 2012, and 2016 but arrived on the refuge in 2001, 2008, and 2015, respectively (unpublished data, John T. Hogg). All three immigrants were rams aged 1–3 years on arrival, and all remained (largely) resident on NBR until their death at ages 11, 6, and 5 years (the latter during the 2016 epizootic). Circumstantial evidence suggests that these natural immigrants originated in one or more populations 40–80 km to the west of NBR (Figure 1) and that this nascent tradition of between‐population movement was established after one or more of three rams translocated to NBR in 1993 later made a breeding migration (Hogg, 2000) from NBR to their natal population and back. Resident adult rams have been observed off‐refuge during fall with low frequency but some regularity since the 1980s. Absent tracking devices and/or detailed study in neighboring populations, the destination and full path of these movements were not determined. Resident bighorn sheep have been observed making a second, qualitatively distinct type of off‐refuge movement (John T. Hogg, Amy Lisk, unpublished data) also with low frequency but with increasing regularity since the 1990s. These movements typically occurred in late April and early May and involved small groups of young (1–2 years) individuals of either sex. They are best described as time‐limited, short‐distance, and age‐specific explorations of the margins of the population home range. In the typical case, animals exited the refuge in small groups along the SE boundary and returned by the same route on the same or next day.

Observation of off‐refuge movements by resident bighorn—whether reflecting longer distance movements between populations or short‐range explorations of local home range—has been opportunistic and may underestimate their true frequency and variety. The perimeter fence clearly limits casual off‐refuge movements—while foraging for example—but it is not a significant barrier to bighorn motivated by some fundamental life‐history concern. We have observed bighorn jump the ≤2 m high perimeter fence with apparent ease and pass under it through holes excavated and maintained by black bears. There is a conflict at NBR, and perhaps elsewhere, between the human perception of bighorn home range (strictly within refuge boundaries) and that of bighorn sheep themselves. Collectively, the observations of off‐refuge movements by resident bighorn and the genetic detections of three natural immigrants suggest that the appropriate view, and context for this case report, is that the NBR bighorn population, at the time of the epizootic and in contrast to 1984, was a semi‐open one with a collective home range exceeding refuge boundaries and linked tenuously on a regional scale with neighboring populations via long‐distance travel corridors. Due to the presence of domestic sheep and goat husbandry on private premises in the vicinity of the refuge, both types of off‐refuge bighorn sheep movements described here carry significant risks of contact with domestic sheep or goats, which may in turn lead to *M. ovipneumoniae* transmission to bighorn sheep and its subsequent carriage into the refuge.

### 2.2 Observational field methods

#### 2.2.1 Pre‐epizootic

Data collected prior to 2016 were used to evaluate host and environmental factors relevant to the most common epidemiologic triad hypotheses for enzootic pathogens: that some form of nutritional stress released a pathogen from immune system control (Miller, Hoberg, et al., 2012). Relevant data include large‐sample measurements of seven female life‐history traits and one variable summarizing annual and seasonal environmental favorability (monthly estimates of actual evapotranspiration, AET, which is an index of net aboveground primary productivity). The seven life‐history traits were lamb birth date, lamb birth weight, average fetal growth rate (birth weight divided by gestation duration), adult fertility, yearling fertility (reflecting age at first reproduction), annual reproductive success (ARS), and female survival spring‐to‐spring. Because the epizootic started mid‐year 2016, we used estimates of AET summed over the first six months of each year as our measure of environmental favorability in 2016 relative to previous years. Field and computational methods for all eight metrics are presented in detail in Hogg et al. (2017).

#### 2.2.2 Epizootic

**FIGURE 2:**
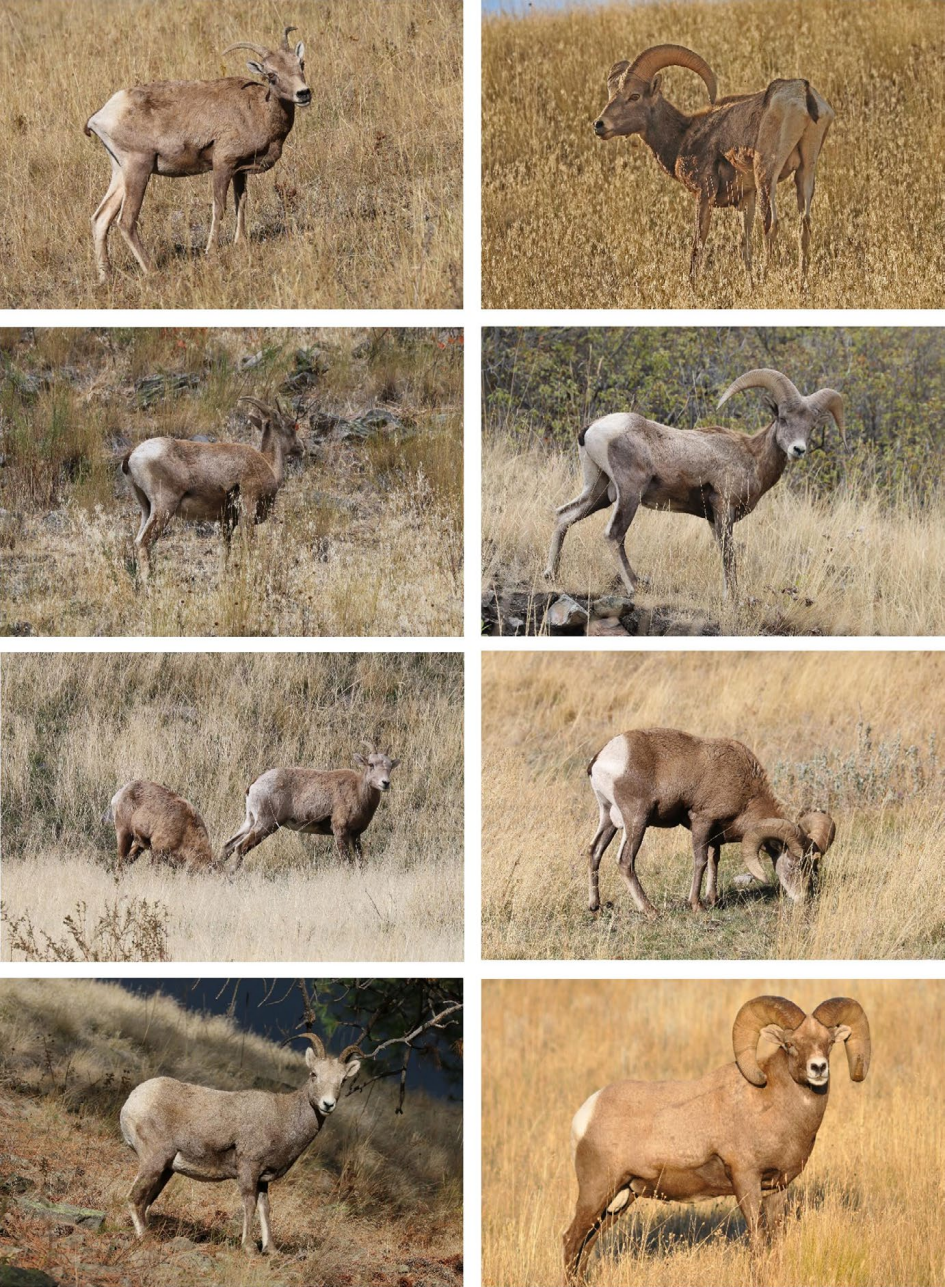
Body condition index (BCI) for exemplar NBR ewes (left column) and rams (right column). Both columns, top to bottom: BCI = 1‐, 2, 3 (photographs from 2016 epidemic), and then BCI = 3 again for an animal photographed in years prior to the epidemic. All photographs taken September–October

We did not anticipate the epizootic. Therefore, field methods specific to the disease event developed over time by modifications of existing study methods. The pre‐epizootic methods included periodic census of both sexes during March/April to determine overwinter survival, daily census of ewe groups during April/May to determine parturition dates, and periodic census during June–October to monitor lamb survivorship. For each group censused, we recorded the date and time of observation, map location, and individual identities of group members when known. Typically, individual identities were assigned by mid‐summer during the second year of life and lambs of the year were always recorded as simple counts. Following onset of the epizootic, we modified these methods as follows:

- Since yearlings (2015 cohort) were not individually recognizable at the onset of the 2016 epizootic or thereafter, we recorded both yearlings and lambs as sex‐specific counts, while continuing to record individual identity of all older animals.
- We increased the frequency of census in the postlambing period to 17 census days in September, 8 in October, 10 in November, and 4 in December. We resumed frequent census in 2017, with 37 census days during the April–July 2017 period. Thus, there were two periods of infrequent and/or poorly distributed census coverage: The first and most significant was July–August 2016 (4 census days clustered during July 30–August 16), and the second was January–March 2017 (3 census days) when weather and other conflicts limited field time.
- During the epizootic, we made a special effort to locate fresh carcasses and assign unambiguous dates of death. This proved difficult in practice largely because social organization dissolved during the epizootic and symptomatic individuals were typically solitary and relatively wary, dispersed widely over sheep range, and often localized in forest or other difficult‐to‐census locations. Absent a fresh carcass, we assigned date of death as the date last seen (alive) in census plus 3 days. Individuals last seen prior to a gap in census of two weeks or longer were assigned a death date at random across the gap assuming a uniform distribution for daily probability of mortality. This included 10 adults (4 females and 6 males) known to be alive in 2016 prior to the epizootic but not seen again. In these cases, date of death was randomly assigned over a period starting 15 July (the earliest date epizootic‐associated mortality could have occurred) and ending 2 September (when regular census resumed).
- We scored individuals for body condition at each census observation in which the animal was standing. Bighorn with a concave, linear, and convex dorsal pelvic plane viewed in profile received coarse condition scores of 1, 2, and 3, respectively (Figure 2). Lesser variations in condition were scored using + or −, where, for example, 1+ and 2‐ both indicated condition in the range >1.0 and <2.0 but closer to 1.0 in the former case and closer to 2.0 in the latter. These scores were recoded numerically to give 8 equally spaced levels of condition: 0.67, 1.0, 1.33, 1.67, 2.0, 2.33, 2.67, and 3.0. To establish a pre‐epizootic sex and date‐specific standard for body condition against which condition during the epizootic could be compared, we scored archival photographs of NBR bighorn using the same condition scale described above. Archival photographs were originally taken for purposes of individual recognition during October–November, and less frequently August–September, between 1996 and 2015, which approximates the range of calendar dates for which epizootic condition scores were available. The only selection requirement was that the photograph captured an individual in full‐body, side‐profile at close range. We allowed repeated measures of photographed individuals in different years and within a year if separated by at least two weeks. Both sexes, all common ages, and females with different reproductive status (± weaned lamb) were represented, as were years with (*n* = 5) and without (*n* = 11) severe drought during the preceding summer.
- Finally, we opportunistically recorded symptoms of bighorn respiratory disease (Besser et al., 2014): prolonged coughing, marked nasal drainage of thick clear or purulent mucus; lethargy (plodding movements and prolonged bedding head down); paresis (drooping) of one or both ears; prolonged nasal licking; repeated head shaking.

### 2.3 Analysis

#### 2.3.1 Pre‐epizootic

We summarized the pre‐epizootic life history and environmental data as follows: First, we calculated a study mean (1979–2016) and annual means for each life history and environmental variable. Annual means based on fewer than four measurements of a trait were dropped. We then computed deviations of the annual trait means from the corresponding study mean and divided each of these by the standard deviation of the trait's annual mean deviations. We did this to put all life‐history and environmental variables on the same measurement scale (number of standard deviations from the study average) for pooled analysis. Because early birth date reflects good condition (Hogg et al., 2017), we reversed sign for birth date so that positive values of the standardized annual mean deviation indicated better than average for all examined variables. To evaluate whether 2016 (or immediately prior years) stood out as stressful in terms of a negative life‐history trait signal, we modeled the standardized annual mean deviations pooled across variables as a function of intercept (fixed effect) and a year‐of‐observation random factor. The standardized annual deviation for each life‐history trait represented a repeat measurement on observation year in this model. Year random effects plus their 95% confidence intervals were extracted using the lmer4 and sjPlot R packages. We evaluated temporal trends in the (standardized) AET index of primary productivity descriptively.

#### 2.3.2 Epizootic

We evaluated seasonal trend in body condition during the epizootic using a linear mixed effects model in which the condition index was modeled as a function of calendar date, calendar date squared, sex, the interaction of date and sex (fixed effect variables), and random factors for both individual slope and intercept. We rescaled calendar date to decimal months beginning 1 September (when condition was first recorded with regularity in 2016). This means, for example, that the main effect of sex in this regression represents an estimate of the difference in average female versus male condition on 1 September, whereas the main effect of calendar date estimates the instantaneous rate of change in condition for the reference sex on 1 September. We estimated main effects of calendar date for both sexes by running each regression twice, once with females and once with males coded as the reference sex. Descriptive field data analyses were implemented using R v4.0.2 and R packages tidyverse v1.3.0, lubridate v1.7.9, visreg v2.7.0, ggpubr v0.4.0, and twosamples v 1.0.0. Parameter estimates for the mixed model regression were obtained using the R package lme4 v 1.1‐23. Confidence intervals for each mixed model parameter were estimated using the lme4 function “confint” specifying the “profile” method of estimation, and *t*‐statistics were computed using the Kenward–Roger method implemented in the R package lmerTest. Because there was much less variation in fall condition during pre‐epizootic years, and most scores fell at the upper limit of the condition index, we used a permutation test (repeated‐measures ANCOVA) implemented with the R package permuco for both models involving these data: that is, those evaluating the effect on body condition (a) of sex and date during the pre‐epizootic period and (b) of period (during vs. before the epizootic). The former model had terms identical to the epidemic period mixed model (above) whereas the latter added a term for period and evaluated the three‐way interaction between period, sex, and date. Finally, because yearlings and lambs were not yet all individually recognizable in the epizootic year, we estimated trend in number for these age classes by calculating the maximum number of each class recorded per census day over rolling windows of four consecutive census days and assigning maximums to the first day of each 4‐day window. We used a negative exponential model and the power curve function DRC.powerCurve from R packages aomisc and drc to fit trend curves to these data.

### 2.4 Field sampling of bighorn sheep and domestic sheep

Bighorn sheep were chemically immobilized using BAM (0.43 mg/kg butorphanol, 0.29 mg/kg azaperone, 0.17 mg/kg medetomidine, Wildlife Pharmaceuticals, Windsor CO) administered by dart gun. Blood samples (10 ml) for serum extraction were obtained by peripheral venipuncture (BD Vacutainer, VWR, Tualatin OR). Nasal mucus was sampled by deep insertion of swabs (BD BBL CultureSwab EZ, VWR) in both left and right nares. Oropharyngeal swab samples were obtained by inserting swabs through oral specula. Nasal swab samples for domestic sheep were obtained during minimal manual restraint of standing animals. Bighorn sheep necropsies were conducted as previously described (Woodford, 1999). Ethical approvals for these animal procedures were obtained from the Washington State University Institutional Animal Care and Use committee (approved protocol #3793) for domestic sheep, and under approved U.S. Fish and Wildlife Service protocols for bighorn sheep.

### 2.5 Microbiology

Detection of respiratory pathogens was performed by the Washington Animal Disease Diagnostic Laboratory, accredited by the American Association of Veterinary Diagnostic Laboratories, using their standard methods. Real‐time polymerase chain reaction was used for detection of *M. ovipneumoniae* on DNA extracted from swab samples from nasal, sinus, or bronchial mucosae, or from lung tissue samples (Manlove et al., 2019). Antibody specific for *M. ovipneumoniae* was detected in blood serum samples using a competitive enzyme‐linked immunosorbent assay (cELISA; Ziegler et al., 2014). Aerobic bacteria were isolated from swab samples of the oropharyngeal mucosa using conventional bacteriologic culture media incubated under aerobic conditions with 5% CO_2_ and subsequently identified by conventional biochemical reactions or by matrix‐assisted laser desorption/ionization (MALDI Biotyper, Bruker, Billerica MA). For a subset of animals with *M. ovipneumoniae* detected by PCR, including the five animals subjected to field necropsy and five additional randomly selected PCR‐positive animals, genetic strains of the pathogen were identified using a 4‐locus multilocus strain typing (MLST) scheme as previously described (Cassirer et al., 2017). For domestic sheep flock strain typing, MLST was conducted in stages: The intergenic spacer (IGS) locus sequence was determined on all swabs in which *M. ovipneumoniae* was detected by PCR, and the other three locus sequences were determined only on those with IGS sequence matching that of the bighorn epizootic strain. DNA sequences of MSLT loci may be accessed in GenBank (MW303758‐303893).

### 2.6 Case report

#### 2.6.1 Pre‐epizootic

**FIGURE 3:**
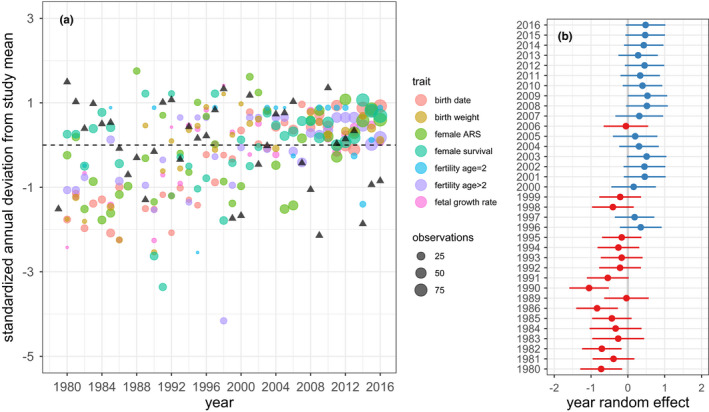
(a) Temporal trend in standardized annual means for seven life‐history traits (circles) and for an index of study site net primary productivity (AET) summed January through June (triangles). We standardized means by calculating the deviation of each annual mean from the corresponding study grand mean and dividing these deviations by the standard deviation of annual means. The *y*‐axis is therefore in units of number of standard deviations from the grand mean. Each point represents one study year and life‐history trait combination. Circle size is scaled by the number of measurements made on a trait in that year. Since early birth date reflects good condition, we reversed sign for birth date so that positive values indicate better than average for all eight variables. All data reflect spring measurements and are therefore immediately pre‐epizootic in the case of 2016. (b) Random year effects plus 95% CIs extracted from a mixed model regression of all 213 standardized annual means shown in Figure 3a (i.e., pooled across all eight variables) on year of measurement. See text for details

**FIGURE 4:**
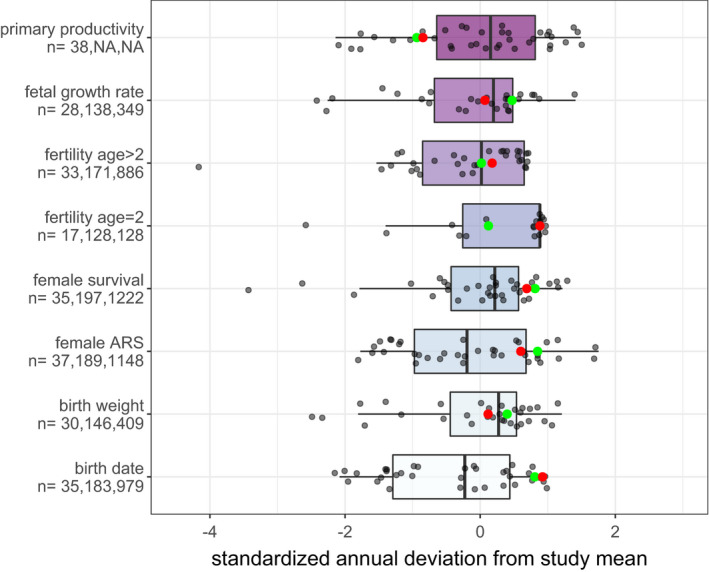
Box‐plot presentation of Figure 3 standardized annual means (circles) for each of seven life‐history traits and for an index (AET) of study site net primary productivity summed January through June. Standardized annual means for the epizootic year (2016) and previous year (2015) are colored red and green, respectively. Sample sizes for each variable are listed on the *y*‐axis. Left to right these indicate number of years contributing data, total number of individuals measured, and total number of measurements, except for primary productivity where only number of years is relevant

The long‐term trend in all seven female life‐history traits was positive (Figure 3), reflecting in large part the previously documented process of genetic rescue (Hogg et al., 2006; Miller, Poissant, et al., 2012). The epizootic year 2016 was no exception to this trend. In fact, the population in this and the preceding year stands out as exceptionally robust phenotypically on an individual trait basis (Figure 4) and in terms of the across‐trait summary metric provided by the year random effect estimates (Figure 3). Confidence intervals on the year random effects suggest that the population during recent years (2008–2016) was not just more robust phenotypically than in many earlier years—none of which were characterized by epizootics—but significantly so. Estimates of AET in the first six months of both 2015 and 2016 were below the study average indicating below average primary productivity. However, the deviation in AET for these years was similarly or less negative than for seven earlier, epizootic‐free years (Figures 3a and 4).

Based on field monitoring conducted April–June 2016, the 30 June population size was 223 individuals: 155 adults (84 ewes and 71 rams), 27 yearlings (15 ewes and 12 rams), and 41 lambs. Clinical signs suggestive of respiratory disease were not observed during this period. Samples from a total of 62 NBR bighorn sheep, obtained between 2006 and April 2016, were tested to detect exposure or infection by respiratory pathogens (Table 1). These included the following: (a) Ten bighorn sheep sampled in 2006 for serology, whose excess serum samples had been stored at −80°C in the interim. (b) Eight bighorn sheep ages 1–2 years (5 male and 3 female) euthanized by CSKT staff in three separate episodes during April and May of 2015 when these animals made off‐refuge exploratory movements. These management actions were based on CSKT policy intended to reduce pathogen spillover by proactively euthanizing bighorn detected during “out‐of‐range” movements. *M. ovipneumoniae* detection was reported from two of the euthanized animals. The *M. ovipneumoniae* were strain typed as BHS‐29, a strain that had been identified since 2011 from bighorn sheep in eight adjacent populations located in the northern Greater Yellowstone Area (GYA), but not elsewhere (Kamath et al., 2019), clearly different from the subsequent NBR epizootic strain. *M. ovipneumoniae‐*positive samples from three bighorn sheep from one of those GYA populations had been shipped to the laboratory in the same container as the samples from the off‐refuge NBR bighorn sheep, raising the possibility of sample cross‐contamination. This possibility was further supported when *M. ovipneumoniae* was not detected in additional lung samples from the same (suspect false positive) animals submitted directly to the diagnostic laboratory. We concluded that the detection of BHS‐29M. *ovipneumoniae* in off‐refuge NBR bighorn sheep were false positives resulting from sample handling error or cross‐contamination. Nevertheless, before the false‐positive status of these tests was clarified, they triggered efforts to assess herd health and reduce population size, as described next. (c) Twenty‐three bighorn sheep were culled during July 2015 for health assessment and necropsy, and 21 residents were translocated in March 2016 to augment the Tucannon and Hall Mountain bighorn sheep herds in eastern Washington State. No evidence of *M. ovipneumoniae* infection or exposure was detected in any of these 44 animals. However, the presence of several other respiratory pathogens was documented, including lungworms (*Protostrongylus* and *Muellerius* spp.), and leukotoxigenic and other pathogenic Pasteurellaceae (*Mannheimia haemolytica* and *Pasteurella multocida*) (Table 1; Besser, Cassirer, et al., 2012; Besser, Highland, et al., 2012; Cassirer et al., 2018; Miller, 2001). The 23 sheep removed from the refuge in 2015 were healthy at necropsy, and no signs of respiratory disease or excess mortality were observed in either of the Washington populations that received NBR animals in 2016 (personal communication, Paul Wik and Annemarie Prince, District Wildlife Biologists, Washington Department of Fish and Wildlife). Samples from the Tucannon population obtained by the WDFW between 2017 and 2019, including several of animals translocated from NBR, showed no evidence of *M. ovipneumoniae* infection or exposure (Table 1).

**TABLE 1:** Microbiological findings from NBR‐associated bighorn sheep prior to, during, and after the 2016 epizootic

Stage	Biological year^a^	Location	*M. ovipneumoniae*		*Pasteurellaceae*	
			cELISA^b^	PCR^b^	LktA PCR^b^	Bacterial culture
Pre‐epizootic^c^	2006	NBR	0 (10)			
Pre‐epizootic^d^	2014	Outside NBR	0 (3)	0.25 (8)^e^		*M. haemolytica, P. multocida*
Pre‐epizootic^f^	2015	NBR	0 (23)	0 (17)	0.63 (16)	*M. haemolytica, B. trehalosi*
Pre‐epizootic^g^	2015	NBR	0 (21)	0 (21)		
Epizootic	2016	NBR		0.906 (32)		
Postepizootic	2017	NBR	0.917 (12)	0.083 (12)		
Postepizootic	2018	NBR	0.737 (19)	0.167 (24)^h^		
Postepizootic	2019	NBR	0.300 (10)^i^	0.0 (10)		
Postepizootic^j^	2017–2019	Tucannon WA	0 (11)	0 (11)		

^a^ Biologic Year: 1 May to 30 April, based on the seasonal life cycle of bighorn sheep.

^b^ Prevalence of detection (*N* animals sampled).

^c^ Banked sera from a serosurvey for malignant catarrhal fever virus, retrospectively tested for *M. ovipneumoniae* antibodies.

^d^ Animals euthanized outside the NBR enclosure to prevent their return, to reduce risk of pathogen introduction.

^e^ The *M. ovipneumoniae* detected in these animals differed from the epizootic strain and are considered likely false positives due to cross‐contamination; see Section 3.

^f^ Animals euthanized within NBR for population management.

^g^ Animals translocated from NBR to Washington State, for NBR population management.

^h^ PCR *M. ovipneumoniae* detections included a single adult ewe and in three 2018 lambs.

^i^ Includes five seronegative 2019 lambs and five adult ewes, three of which were seropositive.

^j^ Includes three translocated NBR bighorn sheep and eight resident Tucannon bighorn sheep.

#### 2.6.2 Epizootic

Clinical signs of respiratory disease were first observed in multiple adult ewes during census 30 July—1 August 2016. A large nursery group observed on 30 June (*n* = 123 sheep, as was typical for that time of year) had fragmented and dispersed. Clinical signs of respiratory disease in adult rams were not observed at this census point. Moreover, rams had a normal grouping pattern (~85% of 71 total adult rams observed in two groups) and were found in seasonally typical locations spatially segregated from ewes.

**FIGURE 5:**
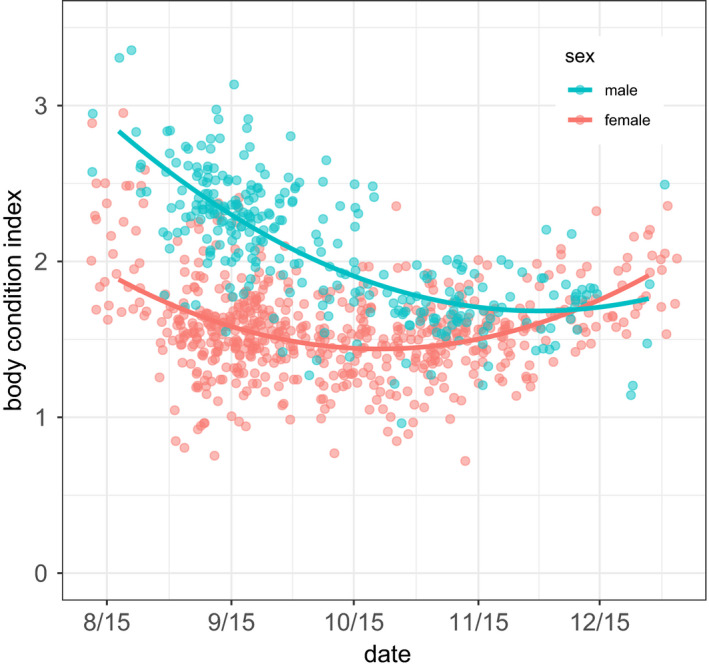
Body condition scores of adult bighorn sheep as a function of calendar date during a pneumonia epizootic at the National Bison Range, Montana, August–December 2016. Symbols are partial residuals, and lines are fitted values from a linear mixed effects model with predictors date, date squared, sex, sex:date (fixed factors), and random factors (intercept and slope) for individual identity. Symbols were jittered vertically and horizontally to reduce overplotting

**FIGURE 6:**
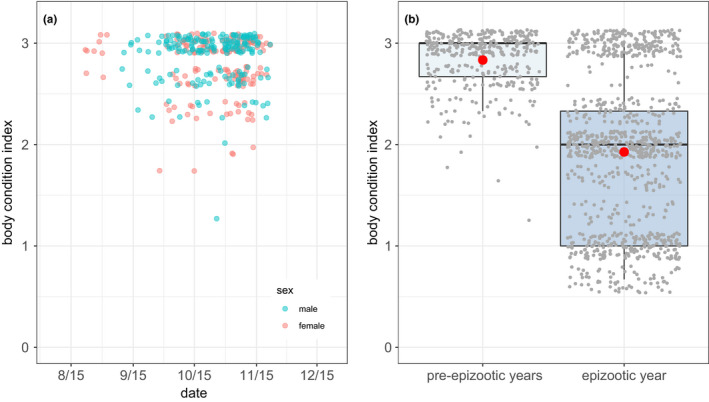
Descriptive statistics establishing a pre‐epizootic standard for the index of body condition (BCI) used during the epizootic. (a) Temporal pattern in fall BCI during pre‐epizootic years for 72 males and 68 females (166 and 188 measurements, respectively). Points were jittered vertically and horizontally to reduce overplotting. (b) Pooled comparison of fall BCI in pre‐epizootic versus epizootic years. Measurements were pooled across individual, gender, and observation date. Red dots indicate mean values; otherwise, both boxplots depict standard metrics (median, interquartile range, highest, and lowest values excluding outliers). Gray dots are jittered individual measurements (354 and 912 measurements on 140 and 138 individuals from pre‐epizootic versus epizootic years, respectively)

When we resumed census and initiated body condition scoring in September, we found that relative to pre‐epizootic standards, the average adult female was in poor condition whereas average body condition of adult males was near normal (Figure 5). This initial difference was substantial, approaching a full unit of the condition index (0.83 units; Table 2). Average condition subsequently trended negative for both sexes but then stabilized or improved later in the year (Figure 5; Table 2). We attribute the latter pattern to both progressive filtering via mortality of animals in relatively poor condition and improved condition among some surviving individuals. During pre‐epizootic years, in contrast, average fall condition was initially high and remained so for both sexes (Figure 6a, Table 3). Considering all data shown in Figure 6b, and for a subset of individuals measured in both time periods, condition during the epidemic was significantly worse than in pre‐epidemic years over the same date range (Table 3).

**TABLE 2:** Effect of calendar date and sex on adult body condition during fall of the epizootic year (2016) as estimated by linear mixed model regression^a^

Predictors	Response: body condition score				
	*B* or *SD*	lCL	uCL	*t*	*p*
Intercept	1.70	1.54	1.86	20.82	<.001
Date (female)	−0.30	−0.37	−0.22	−7.77	<.001
Date (male)	−0.53	−0.63	−0.44	−11.21	<.001
Date squared	0.08	0.07	0.10	10.42	<.001
Sex	0.83	0.58	1.07	−6.63	<.001
Date:sex	−0.24	−0.33	−0.14	4.72	<.001
Random intercept	0.69	0.60	0.78	–	–
Random slope	0.18	0.14	0.22	–	–
Sample sizes	912 measurements on 138 individuals				

Note

Female was the reference sex in all contrasts excepting the main effect of date on male condition in which case sex was reverse coded to make male the reference sex.

^a^ For each fixed effect predictor, we list the regression coefficient (*B*) and likelihood profile lower (lCL) and upper (uCL) 95% confidence limits on *B*. For the intercept and slope random factors, we list the standard deviation of the random effects (intercept and slope) estimated for individual animals and 95% lower and upper confidence limits on the standard deviation. See text for an interpretation of the main effect of sex in the presence of a sex:date interaction and of date with date‐squared in the model.

**TABLE 3:** Repeated‐measures permutation ANCOVA for three models relating body condition to (1) animal sex and calendar date in the pre‐epizootic period; (2) animal sex and calendar date during the epizootic versus pre‐epizootic periods using all measured animals; and (3) as in model 2 but using the subset of animals measured for condition in both periods

Model and terms	SS_N_	*df*_N_	SS_D_	*df*_D_	MSE_N_	MSE_D_	*F*	Permutation *P*(>*F*)^a^
Pre‐epizootic^1^								
Sex	0.006	1	13.56	138	0.006	0.098	0.06	0.81
Date	0.029	1	13.14	138	0.029	0.095	0.30	0.59
Sex:date	0.003	1	13.14	138	0.003	0.095	0.03	0.86
Pre‐epizootic versus epizootic^2^								
Period	13.04	1	350.50	234	13.04	1.50	8.71	0.005
Sex	47.67	1	350.50	234	47.67	1.50	31.82	<0.001
Date	0.03	1	306.80	234	0.03	1.31	0.02	0.88
Period:sex:date	23.13	3	306.80	234	7.71	1.31	5.88	<0.001
Pre‐epizootic versus epizootic^3^								
Period	5.55	1	38.46	39	5.55	0.99	5.63	0.02
Sex	25.20	1	38.46	39	25.20	0.99	25.56	<0.001
Date	0.22	1	32.97	39	0.22	0.85	0.26	0.58
Period:sex:date	20.98	3	32.97	39	7.00	0.85	8.27	<0.001

Note

^1–3^Superscripts denote models 1, 2, and 3, respectively. The effects of factors sex and period were evaluated at the 25th percentile of the date covariate in each model.

^a^ Equals the fraction of 5,000 permutations of the data yielding a *F* statistic greater than or equal to the observed value.

**FIGURE 7:**
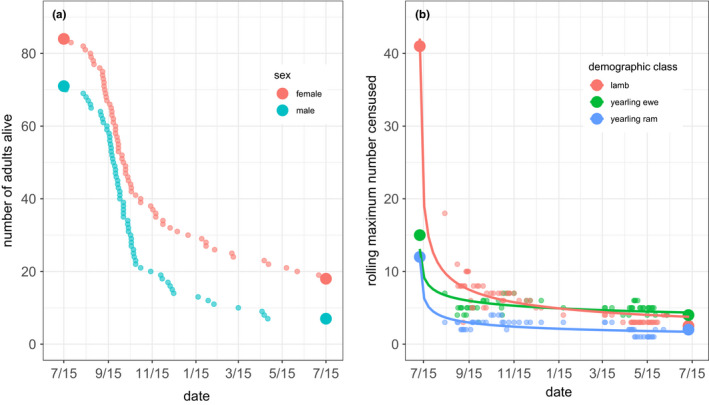
Survival of bighorn sheep 15 July 2016–15 July 2017 following the onset of a pneumonia epizootic at the National Bison Range, Montana. (a) Number of adults (age >two years) alive immediately before (left‐hand large circles), during (small circles), and one year poststart of (right‐hand large circles) the epizootic. Beginning and end numbers are exact counts from census. Numbers between these dates were estimated by subtracting, from the sex‐specific initial total, the cumulative number of individuals dying on or before a given date. (b) Number of lambs and yearlings (age in 2016) alive immediately prior to (left‐hand large circles), during (small circles) and one year poststart (right‐hand large circles) of the epidemic. Beginning and end numbers are exact counts from census. Numbers between these dates are the maximum number of each age/sex class observed per census day over rolling windows of four consecutive census days

**FIGURE 8:**
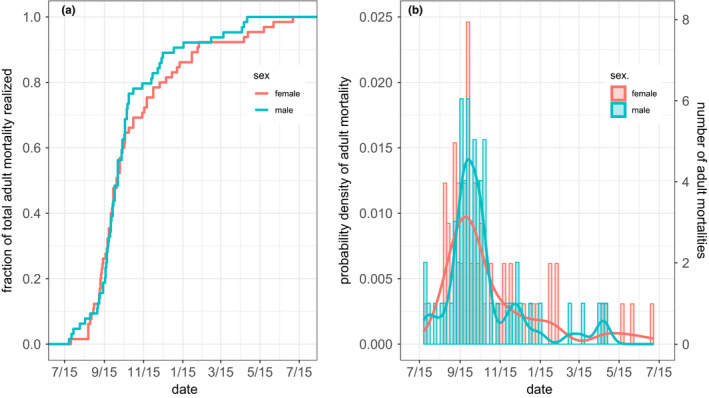
(a) Cumulative probability distribution of adult bighorn sheep mortality during the year following the onset of a pneumonia epizootic at the National Bison Range. (b) Probability density (left axis) and frequency (right axis) of adult mortality (*n* = 133 deaths) during the year following the onset of epizootic‐associated mortality. Dates of mortality were estimated from dates of fresh carcass discovery or, otherwise, dates last seen alive in census

Male and female carcasses were found beginning in early September. However, the pulse in mortality almost certainly started earlier, since ten adult bighorn sheep (4 females and 6 males) known to be alive in spring 2016 were not observed in census after 30 June. By 15 July 2017, approximately one year after the beginning of the epizootic, repeated census revealed that of 223 animals entering the epizootic only 33 survived, a population reduction of 85% (Figure 7a). The 33 survivors included 26 adults (19 females and 7 males), 5 yearlings, and 2 lambs (2016 age classes). Estimates of date of death based on found carcasses and date last seen in census indicate that adult mortality peaked in early October 2016 in both sexes; eighty percent of all adult mortality in the year following onset of the epizootic occurred prior to 31 December 2016 (Figure 8a,b). There was no detectable difference in the timing of adult female versus male death (Cramer–von Mises two‐sample test, *T* = 0.23, *p* = .3). Rolling maximum census counts of lambs and yearlings suggest that mortality in yearlings (both sexes) peaked earlier than in adults with numbers of survivors approaching spring 2017 minimums as early as mid‐September 2016 (Figure 7b) whereas the temporal pattern in lamb mortality more nearly resembled that of adult ewes and rams (Figure 7a,b).

**FIGURE 9:**
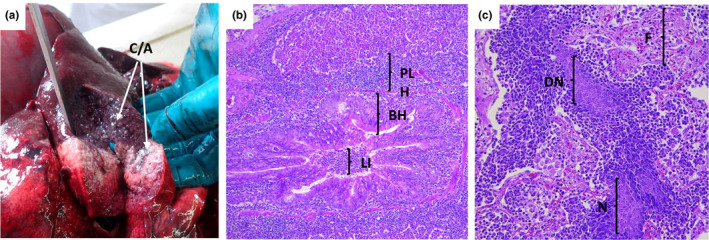
Gross and histological lesions observed in pneumonic NBR bighorn sheep at necropsy. (a) Incision of affected right cardiac lobe reveals consolidation (arrows) due to cellular infiltrates. (b) Histopathology of some affected lung regions reveals bronchiolar epithelial hypertrophy and hyperplasia (BH) with abundant lymphocytic infiltrates (LI) and peribronchiolar lymphatic tissue hyperplasia (PLH), typical of lesions induced by *Mycoplasma spp*. infection. Hematoxylin and eosin. (c) In other regions, pulmonary necrosis (N), fibrin deposition, fibrosis (F), and degenerative neutrophils exhibiting nuclear streaming (DN) are observed, typical of lesions induced by infections with leukotoxigenic Pasteurellaceae or leukotoxigenic *Fusobacterium* spp. Hematoxylin and eosin

During the epizootic, five dead bighorn sheep were subjected to field necropsy. All exhibited anterior ventral lung consolidation typical of bronchopneumonia (Figure 9a). Microscopic lesions included bronchopneumonia, bronchial epithelial hyperplasia, peri‐bronchial lymphoid hyperplasia, and in some sections, pleuritis, pulmonary necrosis, fibrin deposition, fibrosis, and degenerative neutrophils exhibiting nuclear streaming (Figure 9b,c). Bacterial colonies with diverse morphology were observed in affected tissues. Aerobic bacterial cultures of lung tissues detected mixed bacterial infections (Table 1); anaerobic bacteriology cultures were not performed. *M. ovipneumoniae* was detected by PCR in lung tissues and in nasal swabs from all five necropsied animals, as well as in nasal or sinus swabs obtained from 24 of 27 carcasses of bighorn sheep deemed unsuitable for field necropsy due to postmortem scavenging or autolysis. During spring 2017, at least 14 of the 2016 adults (9 ewes and 5 rams) continued to exhibit clinical signs of respiratory disease (chronic coughing and/or marked nasal discharge) as did one male 2016 lamb and one male 2016 yearling (both of whom died prior to the 15 July one‐year anniversary of the epizootic onset).

The *M. ovipneumoniae* from the ten NBR bighorn sheep strain typed by MLST all shared an identical genotype (BHS‐35) that had not been previously detected in either bighorn or domestic sheep (Kamath et al., 2019). With the permission and cooperation of the owner‐operators, nasal swabs from all domestic sheep in the two flocks nearest the NBR were obtained for *M. ovipneumoniae* PCR testing on 29 December 2016 (Flock A, *N* = 66), and on 28 February 2017 (Flock B, *N* = 83). Multiple animals carrying diverse *M. ovipneumoniae* strains were detected in both operations; however, the NBR epizootic strain was not detected in Flock A but was detected in six ewes from Flock B.

#### 2.6.3 Postepizootic

On 28 July 2017, all 33 survivors were scored for body condition. Twenty‐seven scored approximately normal for this time of year (condition index = 3.0) while 6 remained subnormal (mean condition index = 2.2). Only 14 of 23 surviving adult ewes produced lambs in 2017, and all 2017 lambs had disappeared by 60 days after birth. No 2017 lamb carcasses were recovered for sampling or necropsy. However, three of the longest surviving lambs, 34–56 days of age at death, exhibited clear signs of respiratory disease (coughing, ear paresis, nasal discharge, and/or lethargy) prior to disappearance.

Beginning in February 2018, and continuing through April 2020, all surviving bighorn sheep were dart‐anesthetized with the linked objectives of identifying *M. ovipneumoniae* carriers for subsequent removal and preventing recurring epidemics of pneumonia among lambs (Garwood et al., 2020). A single PCR‐positive ewe sampled that first February represented the final *M. ovipneumoniae* detection among adult NBR bighorn sheep. This carrier ewe was present for the period of lambing and rearing in both 2017 (when multiple carriers were likely still alive) and 2018 (when she was the only known carrier). In 2018, *M. ovipneumoniae* was detected (*N* = 3, strain BHS‐35) or indeterminate (*N* = 1) in samples taken from four collected lambs (two collected in May following neonatal predation and two judged to have terminal pneumonia sacrificed in July). Similarly to 2017, none of the 21 lambs born in 2018 survived past 66 days of age and clear symptoms of respiratory disease were observed in six of the seven lambs surviving at least 28 days. The single known *M. ovipneumoniae* carrier ewe was found dead of natural causes in April 2019 just prior to the birth of lambs that year. In marked contrast to 2017 and 2018, clinical signs of pneumonia were not observed for any of the ten 2019 lambs surviving to the age (*ca*. 24 days) at which symptoms typically appear. Seven of the 19 lambs born in 2019 survived to weaning (37%), all of which also survived to one year of age. Finally, five of the seven surviving 2019 lambs were dart‐anesthetized and sampled in April 2020. All five tested *M. ovipneumoniae* negative by both PCR and cELISA, indicating the absence of an active infection and a lack of recent prior exposure to this pathogen (Table 1).

## 3 DISCUSSION

The pneumonia epizootic that struck NBR bighorn sheep in 2016 affected all members of the population, though patterns of morbidity and mortality varied by age and sex. While the epizootic had mostly run its course within 6 months of the onset of symptoms, some clinical signs of respiratory disease and deaths were observed for another 6 months. The 85% population decline associated with the epizootic, precisely determined due to individual animal identification in this closely observed herd, substantially exceeded the 48% median population decline in epizootics across bighorn sheep populations (Cassirer et al., 2018). Over the next two years, evidence of pneumonia was detected in young lambs, all of which died within 10 weeks of birth. In the third year after the epizootic, lamb survival rebounded.

Our results are consistent in every key respect with predictions of the spillover hypothesis: *M*. *ovipneumoniae* was newly introduced into the NBR bighorn sheep population and that this spillover event initiated the 2016 epizootic. These include failure to detect the pathogen prior to the epizootic, detection of a single strain of the pathogen during the epizootic in a pattern consistent with epizootic transmission, identification of a logical route and source for the spillover of the pathogen into the affected population, and continued lamb pneumonia epizootics only so long as adult carriers of the pathogen remained detectable. We note that the predictions of the “spillover” hypothesis are not the predictions (and in fact, would be unlikely coincidences) of any broader “epidemiological triad” hypothesis.

In contrast, we found no evidence of increased susceptibility of the NBR bighorn sheep population due to adverse effects of demographic state or environmental conditions on host nutritional condition, the factor most commonly invoked by the epidemiological triad hypotheses (Miller, Hoberg, et al., 2012). Average birthweight, fetal growth rate, birth date, adult female fertility, age at first reproduction, female reproductive success, and female survival were all stable and at or near study maximums in the epidemic and immediately preceding years. These traits should be particularly sensitive indicators of nutritional stress for several reasons. First, because the 2016 epizootic was so severe, afflicting most individuals in the population, the hypothetical stressor would have to have been similarly broad acting and so likely reflected in population mean performance. Second, each indicator trait is strongly or primarily influenced by spring conditions and therefore should be reflective of maternal physiological condition in the immediate pre‐epizootic period. Third, trait sample size in terms of number of years, individuals, and measurements was sufficiently large to provide reasonable power for detecting signals of physiological stress had such existed. We note that the analysis is completely general with respect to the hypothesized stressor(s); individual nutritional state might decline due to increased population size, reduced forage quality, a combination of these, or something else. The pre‐epizootic population was on every measure available to us a phenotypically robust population on a demographic roll.

Others have previously documented increased risk of epizootic pneumonia associated with proximity to domestic sheep, consistent with the spillover paradigm (Monello et al., 2001; Sells et al., 2015; Singer et al., 2000). On the other hand, retrospective efforts to associate environmental changes with occurrence of pneumonia epizootics in bighorn sheep using indirect measures such as weather have generally identified equivocal or mild negative associations (Monello et al., 2001; Sells et al., 2015). Other field studies that have closely monitored pneumonia epizootics in free‐ranging bighorn sheep have similarly described deaths of animals in excellent condition with no evident forage limitations or similar predisposing factors (Cassirer et al., 1996; Dunbar, 1992). Likewise, in the case of the NBR epizootic, both the weight of the available evidence and parsimony strongly favor the transmission of a pathogen from a reservoir host to a susceptible, non‐adapted host as the most likely cause, consistent with the spillover paradigm.

### 3.1 Causative pathogen

The time series of behavioral observations, lung pathology, and pathogen testing in the NBR population provided evidence that this epizootic was induced by infection with *M. ovipneumoniae,* consistent with previous investigations of pneumonia epizootics in bighorn sheep (Besser, Cassirer, et al., 2012; Besser, Highland, et al., 2012). While several respiratory pathogens (notably not including *M*. *ovipneumoniae*) were detected in NBR bighorn sheep prior to the epizootic, no evidence of pneumonia was observed in the previous 40 years of close monitoring, nor were lesions associated with respiratory disease found in 23 animals culled for comprehensive necropsy in a 2015 health assessment, one year before the epizootic.

Other than what we believe to be the false‐positive “detections” of *M. ovipneumoniae* in two off‐refuge NBR bighorn sheep in the spring 2015 addressed previously, we found no evidence of *M. ovipneumoniae* infection or exposure of the NBR populations prior to the epizootic, including the 2015–2016 period when 52 individuals, representing 23% of yearlings and adults alive at the time were tested (Table 1). Therefore, our data indicate that NBR bighorn sheep were healthy and not exposed to *M. ovipneumoniae* during at least the ten‐year period preceding the epizootic (Table 1) and, further, that the epizootic onset coincided closely with the first detection of *M. ovipneumoniae* in this population.

### 3.2 Source and timing of spillover

Kamath et al. (2019) reported domestic sheep‐origin *M. ovipneumoniae* exhibited very high strain diversity within flocks yet little sharing of strains between flocks: They detected 159 unique strains among 184 positive animals from 60 domestic sheep flocks and herded operations, including 156 and 3 strains that were detected on only 1 and only 2 premises, respectively. Therefore, relevant to seeking the reservoirs that are the source of spillover infections to bighorn sheep: It is unlikely that an epizootic strain will be found in multiple potential source flocks since strain sharing among flocks is rare, but systematic, complete sampling within the source flock may be required to detect the epizootic strain due to the expected high within‐flock prevalence and diversity. Source flock identification is most practical if the number and sizes of plausible sources are relatively small, and the cooperation and assistance of source flock owners is essential. As a result of those limitations, and because the technology for strain typing is relatively recent, detection of presumptive sources of spillover strains to bighorn sheep has only rarely been accomplished: In one case, a single stray domestic sheep present in a Nevada bighorn sheep range during an ongoing pneumonia epizootic carried the same strain of *M. ovipneumoniae* (BHS‐55/DS‐96) implicated in the epizootic (Kamath et al., 2019; unpublished data, Nevada Department of Wildlife). Similarly, in a second case, a single stray domestic goat was found associating with two pneumonic bighorn ewes distant from any identified bighorn range (unpublished data, Nevada Department of Wildlife). Both bighorn sheep and the goat shared a single *M. ovipneumoniae* strain, which belonged to the domestic goat genetic clade of the pathogen (Kamath et al., 2019). Our detection of the bighorn sheep epizootic strain of *M. ovipneumoniae* in domestic Flock B represents an additional observation of a domestic sheep reservoir as the likely source of spillover and for the first time involved a bighorn sheep population with a range partially overlapping that of the candidate source domestic sheep flock. Without pre‐epizootic samples from Flock B, we cannot eliminate the possibility that Flock B domestic sheep and NBR bighorn sheep were infected simultaneously or sequentially (bighorn first then Flock B) by an unidentified third source (wild or domestic). However, the more plausible and parsimonious explanation is that Flock B was the source of the strain of *M. ovipneumoniae* that triggered the NBR epizootic. The residual uncertainty over strain source reflects the relative absence of regional information on strain type diversity and sharing among in domestic sheep and goat operations.

The spillover of *M. ovipneumoniae* to bighorn sheep almost certainly occurred after the 30 March 2016, translocation of 21 healthy, test‐negative animals out of the population and before the first observation of clinical symptoms on 1 August. The lack of field observations of respiratory disease symptoms in repeated census through 30 June, given the approximately 2 week presymptomatic incubation period observed in susceptible bighorn sheep experimentally exposed to *M. ovipneumoniae* (Besser et al., 2014), further narrows the most likely bounds on the index case to 15 June–15 July. This *ca*. 4‐week window is narrower than any previously documented for naturally occurring bighorn sheep pneumonia epizootics (Besser, Highland, et al., 2012; Cassirer et al., 2018).

Spillover risk associated with exploratory movements by bighorn sheep during spring and summer is not widely recognized or reported. Many pneumonia epizootics in mountain sheep occur during fall and winter and are thought to be associated with increased movements of rams during the late fall breeding season (Cassirer et al., 2013; O'Brien et al., 2014). This observation has led to a perception that there is reduced risk of spillover at other times of year, specifically during spring and summer (Borg et al., 2017). However, the timing of the NBR epizootic onset suggests that the bighorn‐domestic sheep *M. ovipneumoniae* transmission event that triggered the NBR epizootic occurred during transient short‐range exploration of the home range margin, likely by young animals (age 1–2 years) in early summer, similar in nature to the off‐refuge movements that motivated the 2015 cull. Alternative explanations (immigration by infected bighorn sheep originating in neighboring populations or domestic sheep movements into core bighorn range) are less likely. The fact that we detected no new nonresident adults during regular census in spring 2016 argues against an adult bighorn immigrant vector. But because resident yearlings were not yet individually recognizable, we cannot rule out introduction from a yearling immigrant. Historically, however, immigration has been much less frequent than spring exploratory forays by residents. Furthermore, the NBR epizootic strain type differed from all those we have previously detected in either bighorn or domestic sheep (Kamath et al., 2019). The domestic sheep operations adjacent to NBR did not report fencing problems or loss of stray animals during this period. Well‐maintained fencing is generally considered an effective barrier to movement of domestic sheep, and domestic sheep have never been observed within refuge boundaries.

The NBR bighorn sheep population was increasing, with a minimum count of 223 just prior to the epizootic. Pneumonia epizootics are reported to be more frequent in larger, growing bighorn sheep populations closer to their peak size, although the mechanism for this relationship is not understood (Monello et al., 2001; Sells et al., 2015). While those studies showed strong tendencies for epizootics to occur shortly after peak bighorn sheep population size or density, neither addressed whether or the degree to which this relationship resulted from direct epizootic‐associated mortality and the prolonged periods of impaired recruitment that commonly follow epizootics (Monello et al., 2001; Sells et al., 2015). We report here that 52 bighorn sheep from the age classes most frequently observed making off‐refuge movements were proactively removed from NBR before the epizootic (Table 1), yet this population reduction did not prevent the exploratory movements that resulted in spillover and the subsequent epizootic.

### 3.3 Dynamics of within‐population pathogen transmission and spread of disease

Two lines of evidence suggest that, following spillover, spread via bighorn‐bighorn transmission radiated through ewe groups before adult ram groups. First, clinical symptoms of respiratory disease were observed in ewe nursery groups at a time when adult rams appeared healthy. Second, while declines in body condition were eventually observed in both sexes, they were more severe in ewes than in rams during the first months of the epizootic. This stepped‐path scenario for transmission is consistent with our hypothesis that the seminal bighorn‐domestic contact derived from a transient spring foray by juvenile bighorn, since yearling rams, two‐year old rams, and yearling ewes were, respectively, generally, often, and almost always associated with female groups in spring. Thus, young bighorn sheep returning from an exploratory foray would most likely join ewe social groups. Deaths also peaked in yearlings first, followed by all other age classes, including lambs. Despite the earlier appearance of clinical signs in ewe groups, the timing of mortality did not differ between sexes, indicating that rams succumbed more quickly following the onset of symptoms.

### 3.4 Timing and mechanism of pathogen clearance

Brief to prolonged periods of low recruitment following all age epizootics due to pneumonia epizootics in young of the year is characteristic of this disease. This signature feature is thought to be caused by the presence of persistent infected but largely asymptomatic carriers that transmit *M. ovipneumoniae* to susceptible lambs (Cassirer et al., 2018; Garwood et al., 2020; Spaan et al., 2021). Postmortem testing of lambs in 2018 confirmed *M. ovipneumoniae‐*associated pneumonia, and death of the single known carrier ewe in spring 2019 prior to the seasonal onset of parturition coincided with the first pneumonia‐free (and serologically *M. ovipneumoniae* exposure‐free) cohort of lambs, suggesting that the presence of one carrier female was associated with the lamb pneumonia epizootic in 2018. Spaan et al. (2021) have reported a similar population response to natural death of a single carrier ewe. The relatively short duration of postepizootic, pneumonia‐induced mortality in NBR lambs could be due to several factors, including the low number of survivors, fitness costs associated with carriage (Dekelaita et al., 2020), or the high virulence of this epizootic selecting against animals less likely to resist infection and thus become carriers.

### 3.5 Future research

This case study raises several yet unanswered questions fundamental to an improved understanding of factors contributing to pathogen spillover to bighorn sheep and the subsequent severity and duration of disease. These include the following: Does spillover of *M. ovipneumoniae* strains directly from domestic sheep and goats produce more severe epizootics than exposure to strains that have become established in other bighorn sheep populations? Is infection less likely to persist via chronic carriers in bighorn sheep populations following severe, high mortality epizootics? How are *M. ovipneumoniae* strains distributed and shared among regional domestic sheep flocks and goat herds, and how definitive is detection of an epizootic strain within a local flock? How does bighorn sheep population size, spatial structure, and demographic composition affect epizootic dynamics including spillover, mortality, and recovery rates? Finally, what is the role of individual and population genetics in determining the course and severity of respiratory disease in bighorn?

## 4 SUMMARY AND CONCLUSIONS

This unanticipated pneumonia epizootic in a well‐studied population provided an opportunity to investigate the causes and short‐term outcome of a major perturbation by an emerging infectious disease. The results have significant implications for understanding and managing spillover in wild sheep. First, the onset and subsequent fadeout of respiratory disease in the NBR bighorn sheep population were very closely tied temporally with the introduction and clearance of *M. ovipneumoniae*, respectively, providing strong support for the proposed primary causal role of this respiratory pathogen. Genetic strain typing of *M. ovipneumoniae* conducted in neighboring wild and domestic sheep populations uniquely resulted in tracing of the spillover pathogen to a specific domestic sheep flock. Second, the epizootic started in summer and was not associated with movements of bighorn sheep during the breeding season, differing from scenarios reported or suspected in many previous pneumonia epizootics. Third, this epizootic occurred despite management actions to significantly reduce the bighorn sheep population, intended to decrease the risk of spillover. Fourth, this epizootic followed dramatically positive responses to the earlier genetic rescue of this population, suggesting that regional variation in population genetics may not be particularly important in providing resistance to a novel spillover pathogen, at least on an ecological time scale. Fifth, no evidence was found to support a role for environmental factors causing or contributing to the epizootic. Finally, this case report adds to the growing evidence that free‐ranging bighorn sheep populations are placed at existential risk by contacts with domestic animal reservoirs of *M. ovipneumoniae*.

## CONFLICT OF INTEREST

This study was supported by funding sources cited in the Acknowledgments. Additional research funding for the authors’ bighorn sheep pneumonia‐related research has been received from the US Department of Agriculture (including the Animal Plant Health Inspection Service and the US Forest Service), the US Geologic Survey, the Federal Aid to Wildlife Restoration program, numerous chapters and affiliates of the Wild Sheep Foundation, and the WSU Fowler Emerging Infectious Diseases Endowment, all listed here because they may represent perceived conflicts of interest.

## AUTHOR CONTRIBUTIONS

**Thomas E. Besser:** Conceptualization (equal); Data curation (equal); Formal analysis (equal); Funding acquisition (equal); Investigation (equal); Methodology (equal); Visualization (equal); Writing‐original draft (equal); Writing‐review & editing (equal). **E. Frances Cassirer:** Conceptualization (equal); Data curation (equal); Funding acquisition (equal); Methodology (equal); Visualization (equal); Writing‐original draft (equal); Writing‐review & editing (equal). **Amy Lisk:** Data curation (equal); Funding acquisition (equal); Investigation (equal); Writing‐review & editing (equal). **Danielle Nelson:** Formal analysis (equal); Investigation (equal); Visualization (equal); Writing‐review & editing (equal). **Kezia R. Manlove:** Funding acquisition (equal); Writing‐review & editing (equal). **Paul C. Cross:** Funding acquisition (equal); Writing‐review & editing (equal). **John T. Hogg:** Conceptualization (equal); Data curation (equal); Formal analysis (equal); Funding acquisition (equal); Investigation (equal); Methodology (equal); Visualization (equal); Writing‐original draft (equal); Writing‐review & editing (equal).

## Data Availability

Raw data used in the preparation of this manuscript are accessible in Dryad (DOI https://doi.org/10.5061/dryad.bvq83bk98). MLST sequence data for *M. ovipneumoniae* have been deposited in GenBank accessions: MW303758–303893.
